# Clinical impact of the 21-gene recurrence score on adjuvant treatment selection in elderly patients with hormone receptor-positive, HER2-negative early breast cancer

**DOI:** 10.1186/s12885-026-15948-w

**Published:** 2026-04-11

**Authors:** Asuka Kochi, Shinichiro Kashiwagi, Kei Nakada, Ayako Gose, Mai Nishimoto, Mariko Nishikawa, Chika Watanabe, Koji Takada, Yukie Tauchi, Kana Ogisawa, Haruhito Kinoshita, Masatsune Shibutani, Tamami Morisaki

**Affiliations:** 1 Department of Breast Surgical Oncology, Osaka Metropolitan University Graduate School of Medicine, 1-4-3 Asahi-machi, Abeno-ku, Osaka, 545-8585 Japan; 2 Department of Gastrointestinal Surgery, Osaka Metropolitan University Graduate School of Medicine, 1-4-3 Asahi-machi, Abeno-ku, Osaka, 545-8585 Japan

**Keywords:** Oncotype DX, Elderly breast cancer, Adjuvant therapy, Chemotherapy de-escalation, Hormone receptor-positive breast cancer

## Abstract

**Background:**

The 21-gene recurrence score (RS) assay (Oncotype DX) has become a cornerstone for guiding adjuvant systemic therapy in early-stage hormone receptor–positive, human epidermal growth factor receptor 2–negative (HR+/HER2−) breast cancer. While randomized trials have established the clinical utility of RS, its real-world application in elderly patients remains controversial, particularly regarding potential age-related bias in treatment decision-making.

**Methods:**

We conducted a retrospective cohort study of 216 consecutive patients with early-stage HR+/HER2 − breast cancer who underwent curative surgery followed by Oncotype DX testing. Patients were stratified by age (elderly, ≥ 65 years; non-elderly, < 65 years). RS distribution and adjuvant treatment patterns were analyzed using both continuous and categorical RS measures. Adjuvant systemic therapy was classified into standard endocrine therapy, enhanced endocrine therapy, and intravenous chemotherapy, and age-related differences were evaluated overall and across RS categories.

**Results:**

Elderly patients demonstrated a significantly lower median RS compared with non-elderly patients (11 vs. 15, *p* = 0.008), with a distinct shift toward lower RS categories (*p* = 0.039). However, substantial overlap in RS distributions was observed, including the presence of high RS tumors among elderly patients. In the overall cohort, adjuvant treatment patterns did not differ significantly by age (*p* = 0.884). When stratified by RS category, no significant age-related differences in treatment selection were observed in low- or intermediate-RS groups. Among patients with high RS, intravenous chemotherapy was administered to both elderly and non-elderly patients without a statistically significant difference (*p* = 0.106).

**Conclusions:**

Although elderly patients exhibited lower RS values overall, tumors with high genomic risk were observed across age groups. Age-related differences in adjuvant treatment selection were attenuated when treatment decisions were interpreted within RS-defined risk categories, including among patients with high RS. These findings support the continued use of genomic assays to facilitate biologically driven and individualized adjuvant treatment decision-making in elderly patients with HR+/HER2 − early breast cancer.

**Supplementary Information:**

The online version contains supplementary material available at 10.1186/s12885-026-15948-w.

## Background

Adjuvant systemic therapy for early-stage hormone receptor–positive, human epidermal growth factor receptor 2–negative (HR+/HER2−) breast cancer has been increasingly individualized through the use of multigene assays [1, 2]. Among these, the 21-gene recurrence score (RS) assay (Oncotype DX) has been widely adopted to estimate recurrence risk and predict the benefit of adjuvant chemotherapy, thereby reducing overtreatment while maintaining oncologic outcomes [1, 2]. The pivotal TAILORx trial redefined RS-based treatment strategies, demonstrating that most patients with low to intermediate RS could safely omit adjuvant chemotherapy, while a subset of patients—particularly younger individuals with higher RS—derive measurable benefit from chemotherapy [3, 4]. Since the publication of TAILORx, RS-guided decision-making has become an integral component of clinical practice, supported by randomized evidence across both node-negative and selected node-positive populations [3–5]. However, the real-world application of RS remains heterogeneous, especially across different age groups [6, 7].

Recent population-based studies in the post-TAILORx era have highlighted age as a major determinant of both RS testing and subsequent chemotherapy use [6, 7]. In large contemporary cohorts, younger patients were more likely to undergo RS testing and receive chemotherapy, whereas older patients underwent testing less frequently and were less likely to receive chemotherapy, even when classified as high-risk by RS [6, 7]. These findings suggest that chronological age may influence clinical decision-making beyond tumor biology alone, and align with geriatric oncology recommendations emphasizing individualized assessment rather than age-based exclusion [8, 9]. Importantly, most prior studies have focused primarily on the binary decision of chemotherapy administration [6, 7]. Less attention has been paid to how adjuvant treatment strategies are modified in older patients, particularly given the heterogeneity of fitness, comorbidities, and competing risks [8–10]. In this context, tools that estimate chemotherapy toxicity risk in older adults have been proposed to guide treatment intensity [11]. Moreover, whether RS-driven risk stratification translates into qualitatively different adjuvant treatment patterns across age groups—such as endocrine therapy intensification or oral agents instead of conventional intravenous chemotherapy—remains incompletely understood [9, 10].

Therefore, in this study, we aimed to evaluate the association between age and RS distribution, as well as age-specific differences in adjuvant treatment selection in patients with early-stage HR+/HER2 − breast cancer. By integrating RS values with detailed categorization of adjuvant therapy—including standard endocrine therapy, enhanced endocrine therapy, and intravenous chemotherapy—we sought to clarify how RS is operationalized in real-world treatment decisions and to identify potential age-related discrepancies in the application of precision oncology [6, 7].

## Methods

### Study design and patients

This retrospective cohort study was conducted at Osaka Metropolitan University Hospital. Consecutive patients with histologically confirmed hormone receptor–positive and human epidermal growth factor receptor 2–negative (HR+/HER2−) invasive breast cancer who underwent curative surgery followed by Oncotype DX testing between September 2023 and July 2025 were eligible for inclusion. Patients were included if complete clinicopathological data—including tumor size, histological grade, lymph node status, and Ki-67 labeling index—were available and a valid Oncotype DX recurrence score (RS) result had been obtained [2, 3]. Patients who received neoadjuvant systemic therapy, presented with recurrent or metastatic disease at diagnosis, or had incomplete clinicopathological or RS data were excluded. A total of 216 patients met the eligibility criteria and were included in the final analysis.

### Clinicopathological variables

Baseline clinicopathological data were extracted from medical records, including age, tumor size, histological grade, nodal status, Ki-67 labeling index, HER2 immunohistochemistry (IHC) status, and clinical risk classification. Tumor size was recorded as the pathological maximum diameter. Nodal status was categorized as node-negative (N0) or node-positive (≥ 1 involved lymph node). HER2 status was assessed by IHC according to standard guidelines [12].

### Oncotype DX recurrence score assessment

The 21-gene recurrence score (RS) assay (Oncotype DX) was performed on formalin-fixed, paraffin-embedded tumor specimens as part of routine clinical practice [1, 2]. RS was analyzed both as a continuous variable and as a categorical variable based on TAILORx-defined thresholds: low (0–10), intermediate (11–25), and high (≥ 26) [3].

### Classification of adjuvant treatment

Postoperative adjuvant systemic therapy was categorized into three clinically relevant groups based on detailed treatment records.

1. Standard endocrine therapy was defined as endocrine therapy alone [13].
2. Enhanced endocrine therapy was defined as endocrine therapy combined with additional systemic agents, including S-1 or abemaciclib [14].
3. Intravenous chemotherapy was defined as the administration of cytotoxic chemotherapy via intravenous routes, with or without concurrent endocrine therapy [3, 12].

Adjuvant treatment patterns were evaluated in the overall cohort, across RS categories, and specifically within the high RS subgroup. In this study, oral fluoropyrimidine-based regimens were categorized as treatment intensification strategies rather than conventional intravenous chemotherapy to reflect real-world clinical practice patterns in Japan.

### Statistical analysis

Continuous variables were summarized as medians with ranges or interquartile ranges (IQRs), as appropriate, and were compared between age groups using the Mann–Whitney U test. Categorical variables were expressed as counts and percentages. For comparisons involving categorical variables with more than two categories, including RS category distribution and adjuvant treatment classification, exact statistical testing was performed using the Fisher–Freeman–Halton test to account for small cell counts. Within each RS category, age-related differences in adjuvant treatment patterns were evaluated using exact 2 × 3 contingency analyses. All statistical tests were two-sided, and a *p* value < 0.05 was considered statistically significant. All analyses were conducted using JMP Pro version 16 (SAS Institute, Cary, NC, USA).

### Ethical approval

This study was approved by the Institutional Review Board of Osaka Metropolitan University (approval number: #2025-045). Given the retrospective design of the study and the use of anonymized data, the requirement for informed consent was waived. All procedures were conducted in accordance with the principles of the Declaration of Helsinki.

## Results

### Patient characteristics

A total of 216 patients with early-stage hormone receptor–positive and human epidermal growth factor receptor 2–negative (HR+/HER2−) breast cancer who underwent curative surgery and subsequent Oncotype DX testing were included in the analysis. Among them, 61 patients (28.2%) were elderly (≥ 65 years) and 155 patients (71.8%) were non-elderly (< 65 years). Baseline clinicopathological characteristics stratified by age group are summarized in Table 1. Elderly patients had a significantly lower median recurrence score (RS) than non-elderly patients (11 [range, 0–52] vs. 15 [range, 0–51], *p* = 0.008). No significant age-related differences were observed in tumor size, histological grade, nodal status, Ki-67 index, HER2 immunohistochemistry status, or clinical high-risk classification. No patient had four or more positive lymph nodes. The distribution of RS categories according to TAILORx thresholds differed significantly between age groups (*p* = 0.039), with elderly patients more frequently classified as having low RS and less frequently classified as having high RS compared with non-elderly patients. High recurrence score tumors were observed across all age groups; however, the use of intravenous chemotherapy remained limited among older patients.

**Table 1:** Baseline clinicopathological characteristics according to age group

Variable	Total (*n* = 216)	Elderly ≥ 65 (*n* = 61)	Non-elderly < 65 (*n* = 155)	*p*-value
Age, years; median (range)	57 (35–89)	74 (65–89)	53 (35–64)	< 0.001
Tumor size, mm; median (IQR)	16 (11–25)	17 (13–29)	15 (11–24)	0.195
Ki-67, %; median (IQR)	14 (5–20)	15 (9–22)	14 (5–20)	0.422
Recurrence Score; median (range)	14 (0–52)	11 (0–52)	15 (0–51)	0.008
Nodal status				0.478
N0	165 (76.4)	49 (80.3)	116 (74.8)	
N+ (≥ 1)	51 (23.6)	12 (19.7)	39 (25.2)	
Histological grade				0.205
1	147 (68.1)	41 (67.2)	106 (68.4)	
2	48 (22.2)	17 (27.9)	31 (20.0)	
3	21 (9.7)	3 (4.9)	18 (11.6)	
HER2 IHC				1.000
0	73 (33.8)	21 (34.4)	52 (33.5)	
1+	90 (41.7)	25 (41.0)	65 (41.9)	
2+	50 (23.1)	14 (23.0)	36 (23.2)	
Clinical high-risk				1.000
Yes	119 (55.1)	34 (55.7)	85 (54.8)	
No	97 (44.9)	27 (44.3)	70 (45.2)	
RS category				0.039
Low (0–10)	78 (36.1)	30 (49.2)	48 (31.0)	
Intermediate (11–25)	105 (48.6)	25 (41.0)	80 (51.6)	
High (≥ 26)	33 (15.3)	6 (9.8)	27 (17.4)	
Adjuvant treatment				0.884
Standard endocrine therapy	163 (75.5)	47 (77.0)	116 (74.8)	
Enhanced endocrine therapy	11 (5.1)	2 (3.3)	9 (5.8)	
Intravenous chemotherapy	42 (19.4)	12 (19.7)	30 (19.4)	

*IQR* Interquartile range, *IHC*, Immunohistochemistry, *RS* Recurrence Score

Sensitivity analyses using alternative age thresholds of 70 and 75 years demonstrated consistent patterns in recurrence score distribution and adjuvant treatment selection (Supplementary Table 1). In particular, the overall tendency toward lower recurrence scores and limited use of intravenous chemotherapy in older patients remained unchanged across these alternative definitions of elderly status.

### Distribution of recurrence score according to age

**Fig. 1:**
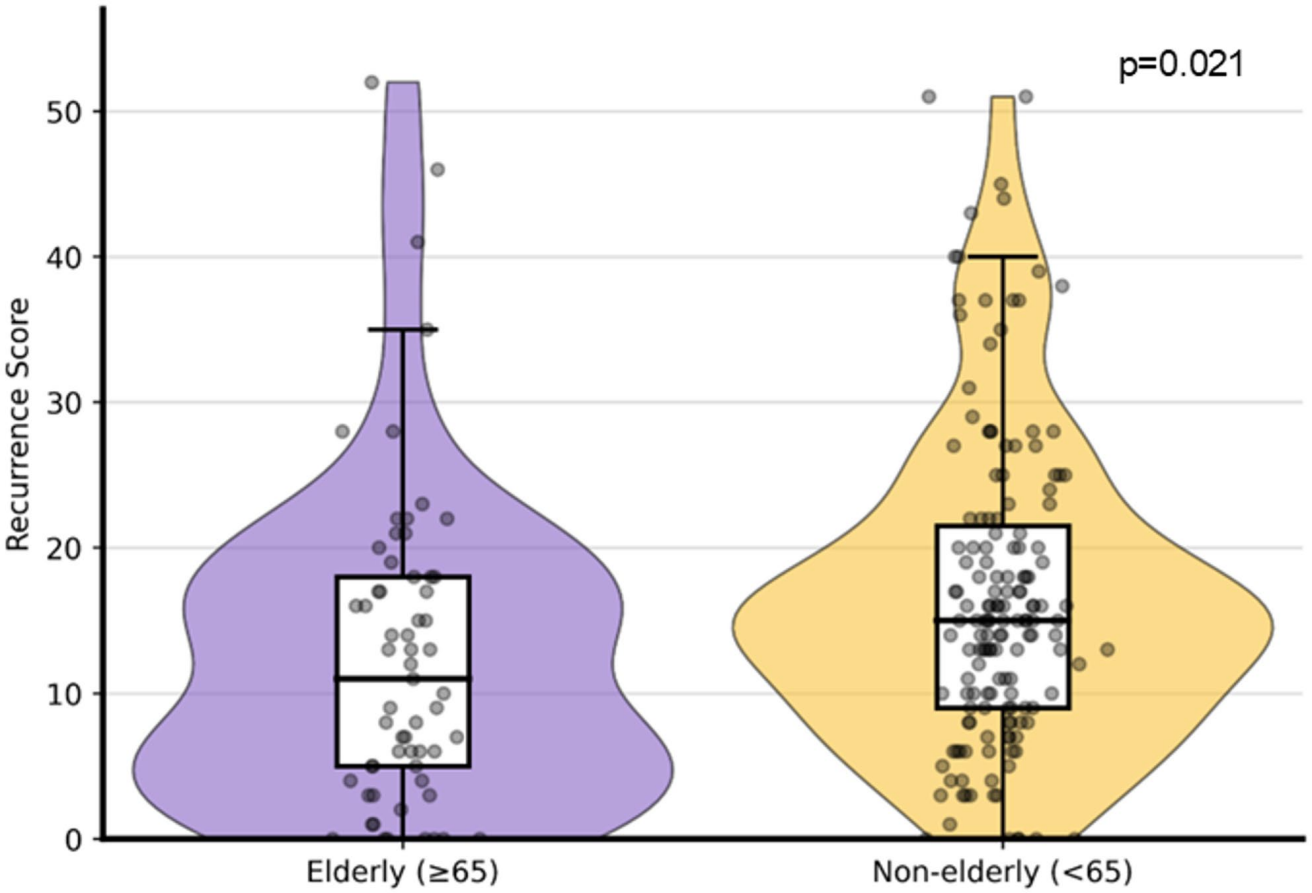
Distribution of Oncotype DX recurrence score according to age group. Violin plots with overlaid box plots show the distribution of recurrence score (RS) values in elderly (≥ 65 years) and non-elderly (< 65 years) patients with early-stage hormone receptor–positive, human epidermal growth factor receptor 2–negative breast cancer. Horizontal lines within boxes indicate median values, and boxes represent interquartile ranges. Elderly patients demonstrated a significantly lower median RS compared with non-elderly patients (*p* = 0.021)

The distribution of RS values according to age group is shown in Fig. 1. Although the median RS was significantly lower in elderly patients (*p* = 0.021), a wide overlap in RS distributions was observed between the two age groups, including the presence of high RS values in elderly patients.

### Adjuvant treatment patterns according to RS category and age

**Fig. 2:**
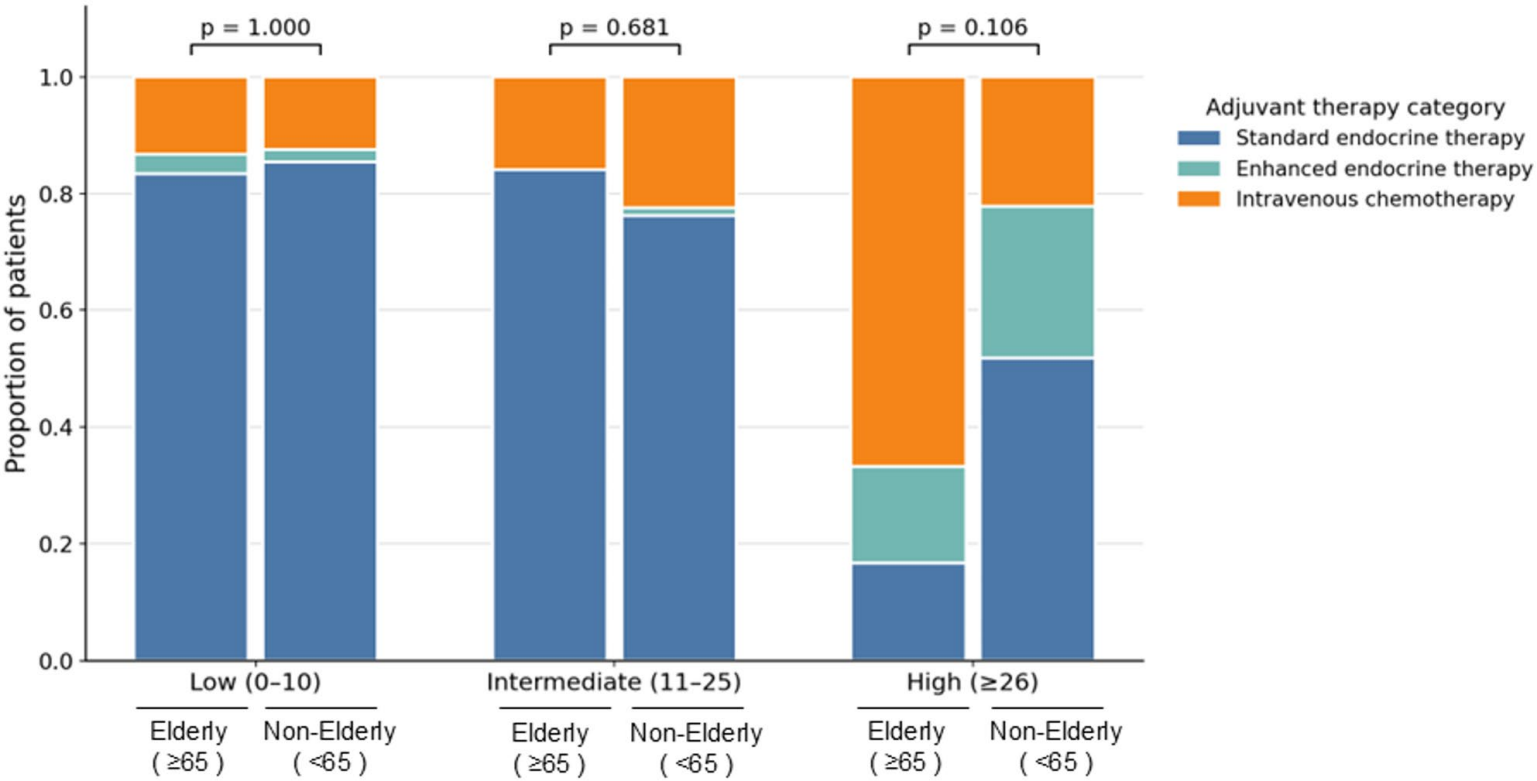
Age-stratified adjuvant treatment patterns according to recurrence score category. Stacked bar plots illustrate the proportions of adjuvant treatment strategies—standard endocrine therapy, enhanced endocrine therapy, and intravenous chemotherapy—according to recurrence score (RS) category (low, intermediate, and high) in elderly (≥ 65 years) and non-elderly (< 65 years) patients. Percentages are shown within each RS category. p values indicate between-age-group differences in the distribution of the three adjuvant treatment categories within each RS category, assessed using exact statistical testing

Adjuvant treatment patterns according to RS category and age are summarized in Table 2 and visually presented in Fig. 2. In the overall cohort, adjuvant treatment patterns did not differ significantly between elderly and non-elderly patients (*p* = 0.884). When stratified by RS category, no significant age-related differences in treatment selection were observed in the low-RS group (*p* = 1.000) or intermediate-RS group (*p* = 0.681). In the high-RS group, intravenous chemotherapy tended to be used more frequently in elderly patients, whereas standard endocrine therapy was more frequently selected in non-elderly patients; however, this between-group difference did not reach statistical significance (*p* = 0.106). Overall, these findings suggest that age-related differences in adjuvant treatment selection were attenuated when treatment decisions were interpreted within RS-defined risk categories.

**Table 2:** Adjuvant treatment patterns according to Recurrence Score category and age group

RS category	Treatment	Elderly ≥ 65 (*n* = 61)	Non-elderly < 65 (*n* = 155)	*p*-value
Low (0–10)				1.000
	Standard endocrine therapy	25 (83.4%)	41 (85.4%)	
	Enhanced endocrine therapy	1 (3.3%)	1 (2.1%)	
	Intravenous chemotherapy	4 (13.3%)	6 (12.5%)	
Intermediate (11–25)				0.681
	Standard endocrine therapy	21 (84.0%)	61 (76.3%)	
	Enhanced endocrine therapy	0 (0.0%)	1 (1.2%)	
	Intravenous chemotherapy	4 (16.0%)	18 (22.5%)	
High (≥ 26)				0.106
	Standard endocrine therapy	1 (16.7%)	14 (51.9%)	
	Enhanced endocrine therapy	1 (16.7%)	7 (25.9%)	
	Intravenous chemotherapy	4 (66.6%)	6 (22.2%)	

*RS* Recurrence Score

## Discussion

In this retrospective cohort study of patients with early-stage HR+/HER2 − breast cancer who underwent Oncotype DX testing, we demonstrated clinically meaningful age-related differences in recurrence score distribution, while also identifying important areas of concordance in RS-guided adjuvant treatment decision-making across age groups. Elderly patients exhibited significantly lower median RS values compared with non-elderly patients, a finding that is biologically plausible given age-associated shifts in tumor phenotype, endocrine responsiveness, and competing-risk considerations in older populations [8–10]. However, despite this overall tendency toward lower genomic risk, high RS tumors were clearly observed among elderly patients, highlighting the substantial biological heterogeneity that persists even in later life. These observations reinforce the concept that chronological age alone does not adequately reflect tumor biology or therapeutic risk–benefit balance, and support the continued clinical utility of genomic assays for individualized treatment planning in older patients rather than reliance on age-based assumptions [8, 9].

Importantly, our findings suggest that recurrence score–guided treatment decision-making may help attenuate age-related differences in adjuvant therapy selection. In the overall cohort, adjuvant treatment patterns did not differ significantly between elderly and non-elderly patients. When stratified by RS category, treatment patterns remained broadly comparable in the low- and intermediate-RS groups, and among patients with high RS, intravenous chemotherapy tended to be used more frequently in elderly patients; however, this difference did not reach statistical significance. These findings indicate that, when genomic risk is explicitly incorporated into clinical decision-making, treatment selection may become more biology-driven and less age-dependent. This interpretation is consistent with evidence from prospective trials showing that the benefit of chemotherapy is largely confined to patients with higher genomic risk and may vary according to age and menopausal status [3–5]. Furthermore, contemporary population-based studies have demonstrated a gradual decline in chemotherapy utilization in HR+/HER2 − early breast cancer following the widespread adoption of genomic assays, while highlighting the persistent influence of non-biological factors such as age on therapeutic decision-making [6, 7].

Another notable finding of the present study was the relatively frequent use of treatment intensification strategies based on endocrine therapy in elderly patients, including the combination of endocrine therapy with oral systemic agents such as fluoropyrimidines. In Japan, such approaches are sometimes considered when conventional intravenous chemotherapy is deemed less suitable because of concerns regarding treatment tolerability, comorbidities, or patient preference. This pragmatic adaptation reflects real-world clinical decision-making, in which therapeutic intensity is tailored not only according to genomic risk but also according to the anticipated balance between treatment efficacy and burden. Our findings therefore highlight the importance of contextualizing genomic assay–guided recommendations within region-specific treatment practices and patient-centered considerations.

These results add nuance to prior population-based studies reporting lower chemotherapy use among elderly patients in the post-TAILORx era [6, 7]. While such studies emphasize age-related disparities in chemotherapy administration, our data suggest that when RS information is available and explicitly integrated into decision-making, treatment selection becomes more biology-driven and less age-dependent, particularly for patients with high genomic risk. In this context, RS appears to function not only as a prognostic and predictive biomarker, but also as a tool that may partially mitigate age-related bias in adjuvant treatment decisions [6–8]. Furthermore, given the well-recognized pattern of prolonged recurrence risk in hormone receptor–positive breast cancer, individualized long-term risk management remains an important consideration regardless of patient age [13, 14]. These consistent findings across alternative geriatric age thresholds further support the robustness of recurrence score–guided treatment decision-making in older patients.

Several limitations of this study should be acknowledged. The retrospective, single-institution design may limit generalizability, and the number of elderly patients with high RS was relatively small, potentially reducing statistical power. In addition, detailed information on comorbidities, functional status, and patient preference—factors that may strongly influence treatment decisions in older adults—was not available [8–11]. Nevertheless, the strength of this study lies in the granular characterization of adjuvant treatment strategies and the use of rigorous exact statistical testing, allowing for a detailed and transparent evaluation of age-related differences in RS-guided clinical practice.

Taken together, our findings suggest that genomic risk stratification using Oncotype DX supports individualized adjuvant treatment selection across age groups and may help ensure that elderly patients with biologically aggressive disease are not systematically undertreated [6–9].

## Conclusions

In conclusion, elderly patients with early-stage HR+/HER2 − breast cancer demonstrated lower median recurrence scores than non-elderly patients; however, high RS tumors were present in both age groups. Although adjuvant treatment patterns did not differ significantly by age in the overall cohort, age-related differences in adjuvant treatment selection were attenuated within RS categories, particularly among patients with high RS. These findings indicate that RS-guided decision-making promotes biologically driven, rather than age-driven, adjuvant treatment selection and supports the continued use of genomic assays to optimize precision oncology for elderly patients.

## Supplementary Information

Supplementary Material 1.

## Data Availability

The datasets generated and analyzed during the current study are not publicly available due to institutional restrictions but are available from the corresponding author upon reasonable request.

## Abbreviations

HR: Hormone receptor
HER2: Human epidermal growth factor receptor 2
RS: Recurrence score
IHC: Immunohistochemistry
IQR: Interquartile range
