# Supplementary material for: Clinical impact of the 21-gene recurrence score on adjuvant treatment selection in elderly patients with hormone receptor-positive, HER2-negative early breast cancer
Source: BMC Cancer. 2026 Apr 11;26:652. doi: 10.1186/s12885-026-15948-w (PMC13191881; doi:10.1186/s12885-026-15948-w)
Supplement: Supplementary file 1 — Supplementary Material 1. [file 12885_2026_15948_MOESM1_ESM.docx]

## **Supplementary Table 1. Sensitivity analysis of baseline clinicogenomic characteristics and adjuvant treatment patterns using alternative age thresholds (≥70 and ≥75 years)**

| **Variable** | **Elderly ≥70**  **(n=35)** | **Non-elderly <70**  **(n=181)** | **p-value** | **Elderly ≥75**  **(n=24)** | **Non-elderly <75**  **(n=192)** | **p-value** |
| --- | --- | --- | --- | --- | --- | --- |
| Age, years;  median (range) | 77 (72–89) | 54 (35–69) | <0.001 | 79.5 (76–89) | 55 (35–74) | <0.001 |
| Tumor size, mm;  median (IQR) | 15 (9.5–24.5) | 16 (11–25) | 0.480 | 15.5 (12.5–23.3) | 16 (11–25) | 0.620 |
| Ki-67, %;  median (IQR) | 10 (9–20) | 14 (5–20) | 0.270 | 10 (8–20) | 14 (5–20) | 0.310 |
| Recurrence Score;  median (range) | 6 (0–52) | 15 (0–51) | 0.004 | 7 (0–46) | 15 (0–52) | 0.011 |
| Nodal status |  |  | 0.190 |  |  | 0.210 |
| N0 | 30 (85.7%) | 135 (74.6%) |  | 21 (87.5%) | 144 (75.0%) |  |
| N+ (≥1) | 5 (14.3%) | 46 (25.4%) |  | 3 (12.5%) | 48 (25.0%) |  |
| Histological grade |  |  | 0.630 |  |  | 0.810 |
| 1 | 23 (65.7%) | 124 (68.5%) |  | 16 (66.7%) | 131 (68.2%) |  |
| 2 | 9 (25.7%) | 39 (21.5%) |  | 6 (25.0%) | 42 (21.9%) |  |
| 3 | 3 (8.6%) | 18 (10.0%) |  | 2 (8.3%) | 19 (9.9%) |  |
| HER2 IHC |  |  | 0.990 |  |  | 0.990 |
| 0 | 12 (34.3%) | 61 (33.7%) |  | 8 (33.3%) | 65 (33.9%) |  |
| 1+ | 15 (42.9%) | 75 (41.4%) |  | 10 (41.7%) | 80 (41.7%) |  |
| 2+ | 8 (22.9%) | 45 (24.9%) |  | 6 (25.0%) | 47 (24.5%) |  |
| Clinical high-risk |  |  | 0.640 |  |  | 0.600 |
| Yes | 18 (51.4%) | 101 (55.8%) |  | 12 (50.0%) | 107 (55.7%) |  |
| No | 17 (48.6%) | 80 (44.2%) |  | 12 (50.0%) | 85 (44.3%) |  |
| RS category |  |  | 0.018 |  |  | 0.028 |
| Low (0–10) | 20 (57.1%) | 58 (32.0%) |  | 14 (58.3%) | 64 (33.3%) |  |
| Intermediate (11–25) | 12 (34.3%) | 93 (51.4%) |  | 8 (33.3%) | 97 (50.5%) |  |
| High (≥26) | 3 (8.6%) | 30 (16.6%) |  | 2 (8.4%) | 31 (16.2%) |  |
| Adjuvant treatment |  |  | 0.006 |  |  | 0.009 |
| Standard endocrine therapy | 33 (94.3%) | 130 (71.8%) |  | 23 (95.8%) | 140 (72.9%) |  |
| Enhanced endocrine therapy | 2 (5.7%) | 9 (5.0%) |  | 1 (4.2%) | 10 (5.2%) |  |
| Intravenous chemotherapy | 0 (0.0%) | 42 (23.2%) |  | 0 (0.0%) | 42 (21.9%) |  |

## IQR; Interquartile range. IHC; Immunohistochemistry. RS; Recurrence Score.
